# Herbal Compounds Play a Role in Neuroprotection through the Inhibition of Microglial Activation

**DOI:** 10.1155/2018/9348046

**Published:** 2018-04-17

**Authors:** Yan Fu, Jianmei Yang, Xingyu Wang, Pin Yang, Yang Zhao, Kun Li, Yongjun Chen, Ying Xu

**Affiliations:** ^1^Department of Physiology, School of Basic Medicine, Shanghai University of Traditional Chinese Medicine, 1200 Cailun Road, Shanghai 201203, China; ^2^Xuhui District Central Hospital, Shanghai 200031, China; ^3^South China Research Center for Acupuncture and Moxibustion, Medical College of Acu-Moxi and Rehabilitation, Guangzhou University of Chinese Medicine, 232 Waihuan Dong Road, Guangzhou 510006, China

## Abstract

Since microglia possess both neuroprotective and neurotoxic potential, they play a crucial role in the central nervous system (CNS). Excessive microglial activation induces inflammation-mediated neuronal damage and degeneration. At present, numerous herbal compounds are able to suppress neurotoxicity via inhibiting microglial activation. Therefore, many researchers focus on pharmacological inhibitors of microglial activation to ameliorate neurodegenerative disorders. Further work should concentrate on the exploration of new herbal compounds, which characteristically inhibit microglial neurotoxicity, rather than modulating neuroprotection alone. In this review, we summarize these herbal compounds, which in the past several years have been shown to exert potential neuroprotective activity by inhibiting microglial activation. The therapeutic targets and pharmacological mechanisms of these compounds have also been discussed.

## 1. Introduction

A large amount of evidence has demonstrated that neuroinflammation plays a significant role in both acute and chronic neurodegenerative disorders including Parkinson's disease, Alzheimer's disease, multiple sclerosis, stroke, and traumatic brain injury (TBI) [1–3]. They are all related to microglial activation and are accompanied by high expression of proinflammatory mediators. Neuroinflammation is a defense mechanism with the purpose of preventing the CNS from being infected and damaged. Microglia, the resident macrophages of the CNS, act as the primary effector cells in mediating neuroinflammation, the activation of which is characteristic of several inflammatory and neurodegenerative disorders [4–7]. Microglia support the normal function of neurons and monitor the health of neurons in homeostasis, the resting state. Therefore, microglia display beneficial effects in normal conditions. Once brain injury or infection occurs, microglia turn into the activated state and secrete a series of proinflammatory and neurotoxic mediators, such as interleukin-1 beta (IL-1*β*), nitric oxide (NO), tumor necrosis factor alpha (TNF-*α*), and reactive oxygen species (ROS), which not only regulate neuronal function and synaptic transmission but also give rise to neuronal oxidative stress and degeneration associated with deficits in a variety of cognitive and memory tasks [8–10]. As mentioned above, activated microglia could cause and regulate the neuroinflammatory reaction by impairing neurons with a mass of proinflammatory mediators that may result in neuronal death in the end.

Thus, protecting neurons through suppressing microglial activation and neuroinflammation may be considered as a potential therapeutic method for improving neurodegenerative disorders. A number of studies proved that natural plants and their active ingredients could ameliorate neurodegenerative progression by suppressing microglial activation and neuronal damage [11, 12]. This article discusses the conclusions about natural compounds, which based on recent studies could prevent neurons from damage via inhibiting microglial activation and neuroinflammation.

## 2. Herbal Compounds

### 2.1. Resveratrol

Resveratrol is a polyphenolic phytochemical that is extracted from plants including grape, peanut, and berry with pharmacological effects on multiple pathological phenomena [13, 14]. Research has shown that resveratrol has bioactivity-containing antioxidative, anti-inflammatory, and anticancer properties and neuroprotection [15–17].

Myeloperoxidase (MPO) plays a very important role in the host defense system against many pathogens. Research has demonstrated that both overactivation and deficiency of MPO result in a pathological state in the brain. However, resveratrol remarkably decreases MPO levels and NO production, which obviously suppressed neuroinflammatory responses including phagocytosis and ROS production in rotenone-triggered microglia. Resveratrol could alleviate the rotenone-induced impaired responses of primary mixed glia from MPO^−/−^ mice. In neuron-glia cocultures, the impairment of neurons could be relieved by resveratrol. The results displayed that resveratrol affected the microglial response to rotenone via modulating MPO and thus prevented neurons from rotenone-induced injury. As mentioned above, the regulation of MPO levels in microglia by resveratrol provide its neuroprotective ability [18]. Resveratrol remarkably improved trigeminal allodynia dose dependently and reduced the high levels of calcitonin gene-related peptide and c-Fos expression in the spinal trigeminal nucleus. In addition, resveratrol inhibited chronic constriction injury-provoked astrocyte and microglial activation and decreased the levels of proinflammatory mediators in the spinal trigeminal nucleus. Moreover, the effect of resveratrol on pain relief was partially regulated via suppressing the phosphorylation of mitogen-activated protein kinases (MAPKs) through the activation of adenosine monophosphate-activated protein kinase [19]. A study demonstrated that in BV2 microglial cell lines, resveratrol could inhibit NLR family pyrin domain containing 3 (NLRP3) activation and IL-1*β* cleavage caused by ATP. In summary, resveratrol could alleviate the deficit of spatial memory in mice with sepsis-associated encephalopathy by suppressing the NLRP3/IL-1*β* axis in microglia [20]. Resveratrol could not only reduce nicotinamide adenine dinucleotide phosphate (NADPH) oxidase-induced level of ROS generation but also alleviate the transposition of the subunit of NADPH oxidase to the cytomembrane caused by lipopolysaccharide (LPS). Furthermore, the effects of resveratrol on neuroprotection were also relevant to the suppression of the activation of MAPKs and nuclear factor-kappa B (NF-*κ*B) signaling pathways in microglia. The study explicitly revealed that resveratrol could prevent dopaminergic neurons from being damaged by LPS, which depended on time and concentration by inhibiting microglial activation and the expression of proinflammatory mediators [21].

The other research has demonstrated that resveratrol treatment in rat with subarachnoid hemorrhage (SAH) could obviously decrease the expression of Toll-like receptor 4 (TLR4), high-mobility group box1 protein, myeloid differentiation factor (MyD88), and NF-*κ*B. In addition, resveratrol remarkably inhibited microglial activation and proinflammatory mediators, which gave rise to the alleviation of neuronal apoptosis, cerebral edema, and behavior deficits at 24 h after SAH [22]. In conclusion, resveratrol is capable of exerting neuroprotection via suppressing microglial activation through the blockage of related pathways, such as the TLR4/MAPK and NF-*κ*B pathways.

### 2.2. Gastrodin

Gastrodin is the primary bioactive component derived from the traditional Chinese herb *Gastrodia elata* Blume root and has been widely used as an anticonvulsant, analgesic, anti-inflammatory, antioxidative, and sedative agent [23, 24].

The research indicated that gastrodin remarkably decreased the levels of proinflammatory mediators such as cyclooxygenase-2 (COX-2), TNF-*α*, inducible nitric oxide synthase (iNOS), and IL-1*β* via blocking the activation of the NF-*κ*B and MAPK pathways in microglia induced by LPS [25]. Li et al. revealed that gastrodin could protect dopaminergic neurons via obviously suppressing microglial activation and the level of IL-1*β*, COX-2, and iNOS in the substantia nigra of rotenone-induced rats with Parkinson's disease [26]. The other research also reported that gastrodin was capable of significantly improving chronic inflammatory pain and the accompanying anxiety-like behaviors in mice induced by complete Freund's adjuvant (CFA). Furthermore, gastrodin treatment could downregulate the increasing expression of glutamate receptor 1, N-methyl-d-aspartate receptor subunit 2A, N-methyl-d-aspartate receptor subunit 2B, and Ca^2+^/calmodulin-dependent protein kinase II-alpha by reducing microglial activation and proinflammatory mediators such as TNF-*α* and IL-6 in the anterior cingulate cortex of mice with CFA injection [24].

Recently, there was also significant evidence indicating that gastrodin elicited strong neuroprotective effects against loss of retinal ganglion cells in an acute glaucoma rat via inhibiting phosphorylated p38 MAPK and the production of proinflammatory mediators in activated retinal microglia. The results demonstrated that gastrodin possessed a potential therapeutic effect on acute glaucoma and other retinal neurodegenerative diseases by suppressing microglial activation [27]. In conclusion, gastrodin is a new drug that could protect neurons through inhibiting microglial activation.

### 2.3. Trans-Cinnamaldehyde

Trans-cinnamaldehyde (TCA) is a main component isolated from the stem bark of *Cinnamomum cassia*, which has been reported to have anti-inflammatory, antioxidative, antibacterial, antifungal, and antiapoptotic properties in a large amount of in vitro and in vivo models [28–30].

A study revealed that TCA could decrease viability loss and apoptosis in neuronal PC12 cells induced by oxygen and glucose deprivation/reperfusion. The effect indicated that TCA could reduce the production of NO. Additionally, using LY294002, the inhibitor of phosphoinositide 3-kinase (PI3K), could abolish the neuroprotection of TCA, demonstrating that the neuroprotection of TCA can be induced via provoking the PI3K pathway [31]. Other studies confirmed that TCA could inhibit LPS-induced inflammation in BV2 cells and reduce the infarction area and neurological deficit score in injured cerebral tissue of mice induced with ischemia/reperfusion. Furthermore, TCA could obviously alleviate neuronal damage by decreasing the levels of iNOS and COX-2 expression through blocking the NF-*κ*B pathway in injured cerebral tissue of mice induced with ischemia/reperfusion. Therefore, TCA may recede neuroinflammation by suppressing microglial activation and play a key role in neuroprotection [32]. Recent research also showed that TCA could promote the degradation of iNOS mRNA in LPS-induced microglia, thus reducing NO production. Additionally, TCA could not only significantly decrease the expression of iNOS and phosphorylated extracellular signal-regulated kinase 1/2 (ERK1/2) in the hippocampus but also evidently alleviate memory deficits and synaptic plasticity damage in LPS-induced mice. They concluded that TCA improved neuronal damage by suppressing microglial activation via degrading the stability of iNOS mRNA [33]. In addition, TCA decreased the expression of iNOS and COX-2 in the LPS-stimulated BV2 cells and noticeably increased the number of tyrosine hydroxylase-positive dopaminergic neurons in the striatum and substantia nigra of mice with 6-hydroxydopamine challenge. These data indicated that TCA has a function of neuroprotection on dopaminergic neurons, which is associated with the suppression of neuroinflammatory responses induced by microglial activation [34]. As mentioned above, TCA has the potential to prevent neuronal damage via inhibiting microglial activation.

### 2.4. Salvianolic Acid B

Salvianolic acid B (Sal B) is the main active ingredient as a water-soluble component of *Salvia miltiorrhiza* roots (Danshen). The studies revealed that Sal B possessed anticancer activity [35, 36]. Other researches confirmed the therapeutic potential of Sal B on hepatic protection, cardiovascular protection, and neuroprotection [37, 38].

In recent research, Sal B could suppress neutrophil infiltration and microglial activation after TBI. Salvianolic acid B could not only reduce the productions of proinflammatory mediators such as TNF-*α* and IL-1*β* but could also upregulate the levels of anti-inflammatory mediators such as IL-10 and transforming growth factor beta 1. These results demonstrated that the neuroprotective role of Sal B on the TBI model may be related to its anti-inflammatory effects [38]. Research has shown that Sal B could reduce the mRNA levels of iNOS, TNF-*α*, and IL-1*β* in LPS-stimulated microglia by decreasing NF-*κ*B activation. Moreover, Sal B could prevent neuronal damage via the inhibition of microglial activation in a coculture system including microglia and neurons [39]. A study demonstrated that Sal B treatment remarkably lessened the infarction volume and neuroinflammation in the middle cerebral artery occlusion rat model. The TLR4/NF-*κ*B pathway could be significantly suppressed by Sal B treatment in the ischemic hemisphere via inhibiting the activation of microglia. Meanwhile, the secretion of IL-1*β* and IL-6 could be decreased by Sal B. This study confirmed that Sal B could significantly alleviate brain damage following cerebral ischemia by inhibiting inflammation in activated microglia [40]. In conclusion, Sal B is a potential herbal compound to improve neuronal damage through inhibiting microglial activation and neuroinflammation.

### 2.5. Tanshinone

Tanshinone is one of the constituents extracted from *Salvia miltiorrhiza* roots, containing tanshinone I and tanshinone IIA. Tanshinone I is one of the critical active ingredients and exhibits many bioactivities, including antioxidative and anti-inflammatory activities in several laboratorial models [41–43]. Research displayed that tanshinone I could destroy the biomembrane reactor in vitro and decrease the bacterial content in vivo [44]. Tanshinone I could protect mitochondria via the nuclear factor erythroid 2-related factor 2-dependent mechanism in SH-SY5Y cells induced by paraquat [45].

Further studies revealed that tanshinone I could significantly decrease the production of several proinflammatory mediators including TNF-*α*, NO, IL-1*β*, and IL-6 and also distinctly inhibit NF-*κ*B activation in activated M1 microglia stimulated by LPS. Furthermore, tanshinone I had the ability to improve motor function, normalize striatal neurotransmitters, and protect dopaminergic neurons in 1-methyl-4-phenyl-1,2,3,6-tetrahydropyridine- (MPTP-) intoxicated mice. The animal studies also revealed that tanshinone I might reduce the increase in TNF-*α* and IL-10 concentrations through modulating microglial activation in MPTP-intoxicated mice. Therefore, tanshinone I has the potential to protect nigrostriatal dopaminergic neurons by decreasing the level of proinflammatory mediators through the inhibition of NF-*κ*B activation in microglia [46].

Tanshinone IIA is also an active constituent of *Salvia miltiorrhiza* and has been widely used for many years in Asia to treat various diseases for its observable organ protective activities [47, 48]. Research showed that MPTP could not only damage nigrostriatal dopaminergic neurons but also induce microglial activation. Western blot and immunohistochemistry revealed that MPTP could increase the expression of NADPH oxidase and iNOS in substantia nigra pars compacta. In addition, the impairment of nigrostriatal dopaminergic neurons and the high expression of NADPH oxidase and iNOS could be reversed by tanshinone IIA treatment. Thus, tanshinone IIA could protect nigrostriatal dopaminergic neurons through suppressing microglial activation and reducing the expression of NADPH oxidase and iNOS in the model of Parkinson's disease [49]. As mentioned above, tanshinones are likely to protect neurons via suppressing microglial activation and reducing neuroinflammation and oxidative stress.

### 2.6. Oxymatrine

Oxymatrine is a major active ingredient isolated from *Sophora flavescens* Ait (kushen), which has been used in China for thousands of years. It has been reported that oxymatrine could exhibit anticancer, antiapoptotic, and neuroprotective effects [50–52].

Oxymatrine could not only reduce the secretion of heat shock protein 60 (HSP60) in BV2 cells stimulated by LPS but also decrease the expression of heat shock factor 1, which is the transcription factor of HSP60. In addition, oxymatrine could alleviate the expression of MyD88, caspase-3, NF-*κ*B, IL-6, iNOS, TNF-*α*, and IL-1*β* in LPS-stimulated BV2 cells. From the results mentioned above, oxymatrine plays a key role in protecting neurons by blocking microglial activation and HSP60/TLR-4/MyD88/NF-*κ*B pathways. Therefore, oxymatrine, herbal compound, represents as a potent therapeutic agent against microglial activation for ameliorating neurodegenerative disorders [53].

### 2.7. Curcumin

Curcumin is a primary ingredient of turmeric, and studies have shown its anti-inflammatory and antioxidative effects, and it observably alleviates CFA-induced pain hypersensitivity [54].

Studies revealed that curcumin could decrease amyloid beta 42- (A*β*42-) induced expression of IL-1*β*, IL-6, and TNF-*α* in microglia, depending on its concentration. Moreover, curcumin showed an effect of inhibiting the levels of phosphor-ERK1/2 and p38 in A*β*42-activated microglia. These results demonstrated that curcumin alleviated proinflammatory mediators released by microglia via inhibiting ERK1/2 and p38 signaling pathways [55]. Curcumin could dramatically ameliorate the phagocytic abilities of prostaglandin E_2_- (PGE_2_-) stimulated N9 cells. Further, curcumin could reverse the decreased effect of PGE_2_ on A*β*42-induced microglial phagocytosis via inhibiting PGE_2_ receptor subtype 2 and protein kinase A signaling pathways [56].

Curcumin inhibited inflammatory response and microglial activation by decreasing the upregulated fractalkine/CX3C chemokine receptor 1, thus protecting neuronal injury in the hippocampal dentate gyrus of fructose-fed mice [57]. Furthermore, the other result demonstrated that using curcumin nanoparticles may be a feasible way of enhancing neurological function in early brain injury of rat following SAH [58]. In conclusion, curcumin is a promising herbal compound to protect neuronal damage in degenerative disorders via suppressing the inflammatory response in microglia.

### 2.8. Other Herbal Compounds

Isobavachalcone is the major constituent extracted from *Fructus psoraleae,* which presents versatile effects including antitumor [59, 60], antibacterial [61], and bone strengthening [62] effects. A study showed that isobavachalcone could recede the LPS-induced oxidative stress and inflammatory cytokine levels and that it possessed an effect of neuroprotection by inhibiting microglia-mediated inflammation [63]. Scutellarin, a member of flavone glucuronide, is considered the major active component of *Erigeron breviscapus* [64, 65]. Scutellarin was able to reduce the distribution of activated microglia and the levels of TNF-*α*, IL-1*β*, and iNOS in vivo. In vitro, it had the ability to prohibit the upregulated level of ROS, NO, and iNOS in LPS-induced BV2 cells [66]. Additionally, many studies demonstrated that scutellarin could regulate the activation of microglia and protection of neurons by the anti-inflammatory effect in primary microglia and BV2 cells [67]. Sophoraflavanone G decreased the cytotoxicity of conditioned medium prepared by activated BV2 cells induced by LPS to PC12 cells and increased cell viability. As mentioned above, sophoraflavanone G was able to suppress neuroinflammation via MAPKs, PI3K/protein kinase B, Janus kinase/signal transducers, and activators of transcription and nuclear factor erythroid 2-related factor 2/heme oxygenase-1 signaling pathways and might react as a potential constituent for various neuroinflammatory conditions [68]. A recent report revealed that four sesquiterpenoids isolated from *Tussilago farfara* also had neuroprotective effects by reducing the levels of NO, PGE_2_, TNF-*α*, and ROS in the LPS-stimulated BV2 cell and PC12 cell coculture system through the blockage of the NF-*κ*B pathway [69]. The other study indicated that treatment with baicalein, a flavonoid from *Scutellaria baicalensis* Georgi, exerted neuroprotective effects on dopaminergic neurons by decreasing TNF-*α*, NO, and superoxide productions in the neuron-glia coculture system with LPS stimulation and blocking morphological change of microglial activation [70]. In addition, Wogonin, another flavonoid from the root of *Scutellaria baicalensis* Georgi, also had a potent neuroprotection by suppressing microglial activation through the blockage NF-*κ*B pathway in vivo and in vitro experiment [71]. These results suggest that a number of natural compounds have the potential to protect neurons via inhibiting microglial activation. Further, it is possible for these natural compounds to be used as therapeutics for neurodegenerative disorders with neuroinflammation.

## 3. Conclusions

**Figure 1:**
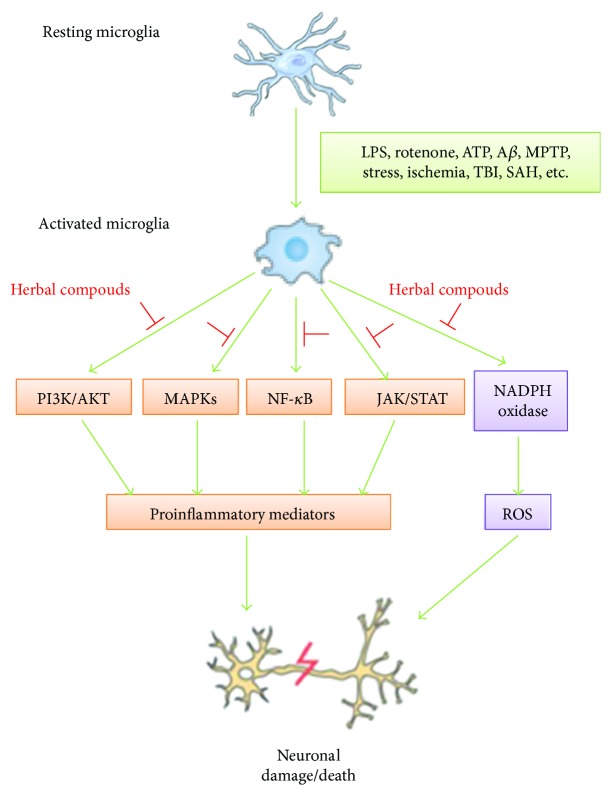
Schematic diagram represents prevention of neuronal damage/death by herbal compounds via inhibiting microglial activation through the blockage of related signaling pathway. LPS: lipopolysaccharide; A*β*: amyloid beta; MPTP: 1-methyl-4-phenyl-1,2,3,6-tetrahydropyridine; TBI: traumatic brain injury; SAH: subarachnoid hemorrhage; PI3k/AKT: phosphoinositide 3-kinase/protein kinase B; MAPKs: mitogen-activated protein kinases; NF-*κ*B: nuclear factor-kappa B; JAK/STAT: Janus kinase/signal transducers and activators of transcription; NADPH oxidase: nicotinamide adenine dinucleotide phosphate oxidase; ROS: reactive oxygen species.

An increasing amount of findings has demonstrated that microglial activation and neuroinflammation play a crucial role in the pathogenesis of neurodegenerative disorders. Recent researches revealed that there are many compounds isolated from natural plants that can delay the neuronal damaged and degenerative progression by inhibiting microglial activation, so they have attracted considerable attention as pharmacological intervention against neurodegenerative discords with neuroinflammatory condition. As illustrated in the summary diagram (Figure 1), these literatures provide the evidences that herbal compounds can protect neuronal damage characterized by neuroinflammatory and oxidative stress condition and they accomplish their role by suppressing microglial activation and proinflammatory and neurotoxic mediator expression via blocking the related signaling pathway in activated microglia. Moreover, the natural products and compounds are inexpensive, easily accessible, and safe. Therefore, they can be widely observed in laboratory researches. If these activities of herbal compounds that are found in laboratory research are beneficial to delaying the development of neurodegenerative disorders, then large and well-designed studies in clinic are required to confirm whether or not their activity is also possible in humans. Further study of the pharmacological mechanisms of natural herbal compounds on the inhibition of microglial activation and neuroinflammation could not only benefit the discovery of effective neuroprotective components but also help researchers to learn more about the pathological mechanisms of neurodegenerative disorders.
